# Effect of Encapsulation of *Lactobacillus casei* in Alginate–Tapioca Flour Microspheres Coated with Different Biopolymers on the Viability of Probiotic Bacteria

**DOI:** 10.1021/acsami.4c10187

**Published:** 2024-09-20

**Authors:** Anna Łętocha, Alicja Michalczyk, Małgorzata Miastkowska, Elżbieta Sikora

**Affiliations:** † Faculty of Chemical Engineering and Technology, Cracow University of Technology, 31-155 Cracow, Poland; ‡ Lukasiewicz Research Network—Institute of Industrial Organic Chemistry, 03-236 Warsaw, Poland

**Keywords:** microspheres, tapioca flour, alginate, probiotics, biopolymers, Lactobacillus casei

## Abstract

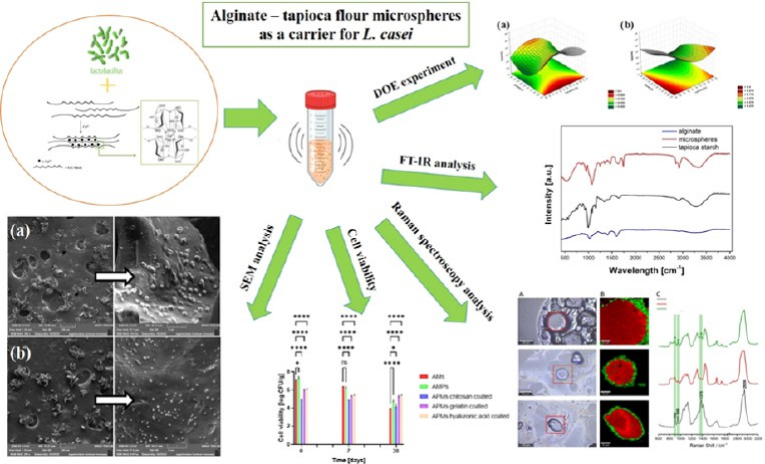

To realize the health benefits of probiotic bacteria, they must withstand processing and storage conditions and remain viable after use. The encapsulation of these probiotics in the form of microspheres containing tapioca flour as a prebiotic and vehicle component in their structure or shell affords symbiotic effects that improve the survival of probiotics under unfavorable conditions. Microencapsulation is one such method that has proven to be effective in protecting probiotics from adverse conditions while maintaining their viability and functionality. The aim of the work was to obtain high-quality microspheres that can act as carriers of *Lactobacillus casei* bacteria and to assess the impact of encapsulation on the viability of probiotic microorganisms in alginate microspheres enriched with a prebiotic (tapioca flour) and additionally coated with hyaluronic acid, chitosan, or gelatin. The influence of the composition of microparticles on the physicochemical properties and the viability of probiotic bacteria during storage was examined. The optimal composition of microspheres was selected using the design of experiments using statistical methods. Subsequently, the size, morphology, and cross-section of the obtained microspheres, as well as the effectiveness of the microsphere coating with biopolymers, were analyzed. The chemical structure of the microspheres was identified by using Fourier-transform infrared spectrophotometry. Raman spectroscopy was used to confirm the success of coating the microspheres with the selected biopolymers. The obtained results showed that the addition of tapioca flour had a positive effect on the surface modification of the microspheres, causing the porous structure of the alginate microparticles to become smaller and more sealed. Moreover, the addition of prebiotic and biopolymer coatings of the microspheres, particularly using hyaluronic acid and chitosan, significantly improved the survival and viability of the probiotic strain during long-term storage. The highest survival rate of the probiotic strain was recorded for alginate–tapioca flour microspheres coated with hyaluronic acid, at 5.48 log CFU g^–1^. The survival rate of *L. casei* in that vehicle system was 89% after storage for 30 days of storage.

## Introduction

According to the generally accepted definition established by FAO and WHO, “probiotics are live microorganisms that, when administered in appropriate amounts, provide the host with health benefits.”^1,2^ The most common probiotics are bacteria in the genera *Lactobacillus* and *Bifidobacterium*. The International Scientific Association for Probiotics and Prebiotics (ISAPP) defines prebiotics as “nondigestible food ingredients that, when consumed in sufficient amounts, selectively stimulate the growth and/or activity of one or a limited number of microbes in the colon resulting in documented health benefits.”^2,3^

The combination of probiotics and prebiotics results in symbiotic activity that improves the survival of bacteria in unfavorable conditions, for example, in the stomach. The most well-known prebiotic is inulin.^1^ Another well-studied prebiotic is resistant starch (hi-maize). Starch is a common, biodegradable, nontoxic, edible, and relatively inexpensive material.^4^ Several reports describe the use of starch to encapsulate food ingredients^5,6^ and drugs.^7,8^ Starch granules are an accumulation of many starch molecules that consist of linear amylose and highly branched amylopectin.^9,10^ The higher content of amylose in tapioca flour (17–23%) may be responsible for the more efficient encapsulation of active substances.^4,10^

However, to achieve the health benefits attributed to the use of probiotics (both in food and cosmetic products), the live bacteria must withstand processing and storage conditions and remain viable after application. In combination with prebiotics, microencapsulation has been shown to be an effective method to protect probiotics from these adverse conditions while maintaining their viability and functionality.^11^ Additionally, in the case of cosmetic products, the use of microcapsules or microspheres offers a solution to the incompatibility of substances when using different active substances in a single formula.^12^ In addition, microparticles enable the encapsulation of substances with an unpleasant odor, and the obtained formulations have a weak or no odor. When probiotics are used in cosmetic formulations, the microencapsulation process may support the survival of microorganisms despite the presence of preservatives.^11,13^ The beneficial properties of probiotics are still underestimated and underutilized in the cosmetics industry. For this reason, our research is focused on the possibility of designing functional microspheres as carriers of probiotic bacteria with potential applications in cosmetic products dedicated to people with skin problems. Restoring the balance of the bacterial microflora will translate into the proper functioning of the microbiome that can promote skin immunity and provide host defense, including protection against skin inflammation, infections, wounds, and skin cancer.^14^

Emulsification is one of the most common microencapsulation techniques.^15^ In this technique, the polymer solution containing the encapsulated substance is emulsified in the oil phase to form a water-in-oil (W/O) emulsion. Thereafter, a cross-linking compound is gradually added to the emulsion, causing the emulsion droplets to gel and form microparticles.^11,16^

Therefore, the aim of this study was to obtain high-quality microspheres through an emulsification process and to evaluate the effect of *Lactobacillus casei* encapsulation on the viability of probiotic bacteria in alginate–tapioca flour microspheres and microspheres coated with various biopolymers including hyaluronic acid, chitosan, and gelatin.

## Materials and Methods

### Materials

Alginic acid sodium salt from brown algae, MRS broth, de Man–Rogosa–Sharpe (MRS) agar, and sodium citrate were purchased from Sigma-Aldrich (Poland). Tapioca flour was purchased from Green Essence (Poland). Calcium chloride was purchased from Avantor Performance Materials Poland S.A. Caprylic/capric triglycerides and ECO-Tween 80 were kindly supplied by Croda (Poland). Chitosan (85% deacetylation) and gelatin (type I) were purchased from Sigma-Aldrich (Poland). Hyaluronic acid sodium salt (0.05–0.1 MDa) was kindly supplied by Alfa Sagittarius (Poland). The probiotic bacteria *L. casei* strain ATCC 393 was purchased from American Type Culture Collection.

### Preparation of the *L. casei* Suspension for Encapsulation

A 250 μL aliquot of the bacterial suspension was transferred into 25 mL of MRS broth contained in an Erlenmeyer flask and incubated at 30 °C under aerobic conditions for 48 h. The cells were harvested when the suspension was in the logarithmic phase by centrifugation at 3000*g* for 10 min at 4 °C. The collected cells were washed twice with sterile saline (0.9%) (w/v). The cell pellets were resuspended in saline solution to obtain concentrations ranging from 9 log CFU/mL to 10 log CFU mL. The bacterial cell count of the cell suspension was determined by counting the cells on plates in MRS agar (37 °C, 72 h culture) using the pour plate inoculation technique.^17^ The obtained bacterial suspensions were used for the microencapsulation procedure.

### Assessment of the Effect of Tapioca Flour Concentration on the Growth of the *L. casei* Strain

This assessment study was based on the methodology of Shafizadeh et al., with slight modifications.^18^ To determine the optimal concentration of tapioca flour required to encapsulate *L. casei*, an appropriate amount of flour was put into liquid MRS to obtain relevant concentrations: 0.5, 1, 2, 4, and 5%. MRS broth without any flour was used as the control. All media prepared in 25 mL quantities in 50 mL Erlenmeyer flasks were sterilized at 121 °C for 15 min in an autoclave. After cooling, the media were inoculated with 1 mL of 48-h *L. casei* culture and incubated for another 48 h at 37 °C under aerobic conditions. *L. casei* bacteria were counted by a serial 10-fold dilutions in sterile saline (0.9%) (w/v). Next, the live bacteria cell count in the medium was determined by counting the cells on plates in MRS agar (37 °C, 72 h culture) using the pour plate inoculation technique. Moreover, after growing the *L. casei* strain on liquid medium with flour, the pH of the culture medium was determined using a pH meter (Metler Toledo). All tests were performed in triplicate, maintaining the principles of sterility.

### Encapsulation of *L. casei*

The encapsulation of *L. casei* was performed in accordance with the modified methodology described by Łętocha et al.^13^ and in the patent application P. 443812.^19^ Briefly, a pre-emulsion was prepared by mixing the encapsulating material (sodium alginate solution, prebiotic solution, and bacterial suspension) with capric/caprylic triglycerides and emulsifier (ECO-Tween 80). An aqueous solution of calcium chloride was added dropwise to the solution to cross-link the microspheres. Then, the obtained microspheres were separated from the emulsion by centrifugation (EBA 20, Hettich Zentrifugen). All solutions used for encapsulation were previously sterilized.

### Optimization of Composition

The composition of microspheres was optimized using mathematical methods (Statistica ver. 13, StatSoft, Poland). To develop the best parameters for the alginate microspheres (AMs) recipe, the design of experiments (DOE) statistical method with the 3^(*K*–*p*)^ fractional plan was used. In this plan, *K* is the number of variables, and *p* always takes the value 1. First, the variables influencing the properties of the final alginate microspheres were determined. The group of input parameters included the amount of emulsifier, the concentration of the prebiotic and alginate solutions, and the mass ratio of alginate to prebiotic. The ranges of the process-independent variables are listed in Table 1.

**Table 1:** Ranges of Process-Independent Variables

independent variable	the ranges of variability
emulsifier concentration (%)	1, 2, 3
concentration of tapioca flour (prebiotic) solution (%)	2, 4, 6
concentration of alginate solution (%)	2, 3, 4
mass ratio alginate to prebiotic (%)	1, 2, 3

### Determination of Microdispersion Droplet Size

To determine the size of the microspheres obtained, the microdispersion was analyzed using an optical microscope before centrifugation. For this test, a small amount of the microdispersion was placed on a glass slide and covered with a coverslip. Observations were made using a Motic B1 Advanced Series microscope equipped with a digital camera. The average ± standard deviation (SD) droplet diameter for each sample was determined on the basis of 200 measurements.

### Viscosity Measurements

The rheological properties of the obtained formulations were determined using a rotational rheometer (Brookfield Model R/S Plus) at room temperature (25 °C), with a shear rate of up to 500 s^–1^, over 60 s.

### Coating of Alginate Microspheres

The alginate microspheres were coated with chitosan, gelatin, or hyaluronic acid. The pH values of the coating solutions were checked using a Mettler Toledo Seven Easy pH meter equipped with a glass Inlab 410 electrode. The alginate microspheres were coated in accordance with the methodology of Krasaekoopt et al.^20^ and Zanjani et al.^21^ Briefly, microspheres (10 g) were immersed in 100 mL of each sterile solution (previously autoclaved at 121 °C for 15 min), coated by stirring for 40 min using a magnetic stirrer, separated by filtration, and rinsed with deionized water.

### Scanning Electron Microscopy (SEM) Analysis

The morphology and cross-section of the obtained microspheres were observed using a scanning electron microscope (Mira3-FEG-SEM, Tescan, Brno-Kohoutovice, Czech Republic) with a one-pole emission (Schottky emitter) equipped with an X-ray energy dispersive spectrometer EDX (Oxford Instruments) and a cooling table (Peltier), operated at a temperature of −30 °C. The samples for SEM investigations were prepared by rapid freezing in liquid nitrogen followed by freeze-drying for 24 h.^22−24^

### Fourier-Transform Infrared (FTIR) Analysis

To identify the chemical structure of the microspheres, attenuated total reflection Fourier-transform infrared (ATR-FTIR) spectrophotometry analysis was performed using a Thermo Scientific Nicolet iS5 FTIR spectrometer equipped with an iD7 ATR accessory. The lyophilized microspheres were meshed and mounted onto the ATR crystal for analysis. The infrared spectra were recorded at wavelengths between 400 and 4000 cm^–1^.

### Raman Spectroscopy Analysis

To confirm the success of the biopolymer coating, Raman spectroscopy was used. All Raman measurements were performed by using a WITec α 300R spectrometer equipped with a confocal microscope, TrueSurface attachment, and charge-coupled device (CCD) detector. For this purpose, a laser line with excitation at a wavelength of 532 nm, an air lens with a magnification of 50×, and a numerical aperture of 0.75 were used. The radiation power at the focus point was 15–17 mW, and the spectral resolution of the collected spectra was approximately 4 cm^–1^. For single spectra recorded for standards and uncoated microspheres, the following parameters were used: number of accumulations, 20; accumulation time for one spectrum, 0.5 s. In each case, three spectra were recorded and then averaged within a given sample.

### Assessment of the *L. casei* Strain Viability and the Effectiveness of Its Encapsulation

To determine bacterial viability (spheres AMs, APMs, and APMs coated with various substances) and the encapsulation effectiveness (spheres APMs in the DOE experiment), a previously described method was used.^13^ To count trapped bacteria, 1 g of spheres was added to 9 mL of 2% (w/v) sterile sodium citrate at a concentration of 0.2 mol/L (pH 6.0), and the mixture was stirred for 10 min. Next, serial dilutions were prepared, and the obtained solutions were inoculated using the pour plate method (1 mL) on MRS agar plates. Viable cells were counted from the number of colonies after being incubated for 72 h at 37 °C under aerobic conditions. All tests were performed in triplicate, maintaining the principles of sterility. The results are presented as the logarithm of colony-forming units per gram (log CFU g^–1^) and as a survival percentage.

The microencapsulation efficiency (EE) (%) of *L. casei* was calculated from eq 1 and presented as the percentage log CFU g^–1^.^25^
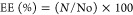
1In the formula, *N* stands for the number of bacterial cells trapped in the microspheres and No stands for the number of free *L. casei* cells added during encapsulation.^18,25^

### Statistical Analysis

All data concerning the mean droplet size and viscosity of formulations, assessments of the effect of flour concentration on the growth of the strain, and the viability of the *L. casei* strain in microspheres were presented as the mean of three different experiments ± SD. The differences between the calculated means of each individual group were determined by one-way analysis of variance (ANOVA) tests, using the statistical software Statistica Version 12 StatSoft Company (Kraków, Poland), and GraphPad Prism. A value of *p* < 0.05 was considered statistically significant.

## Results and Discussion

### Effect of Various Concentrations of Tapioca Flour on the Growth of the *L. casei* Strain

**Figure 1:**
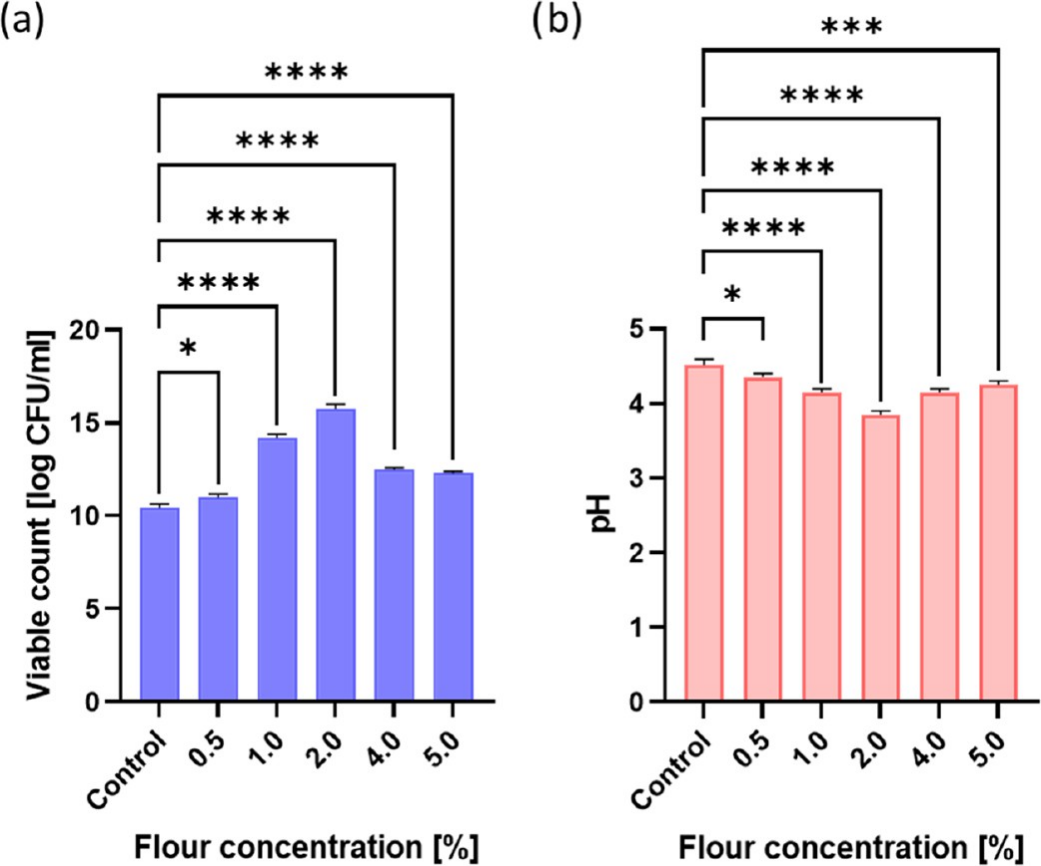
Viable count (log CFU/mL) and pH of *L. casei* values ((a, b) respectively) of the samples containing different concentrations of tapioca flour (error bars represent the SD of the related data). *p = 0.05–0.011; **p ≤ 0.01; ***p ≤ 0.001; ****p ≤ 0.0001.

The effects of various concentrations (0.5, 1, 2, 4, and 5%) of tapioca flour on the growth of the *L. casei* strain and pH changes during MRS broth fermentation are presented in Figure 1a,b, respectively. An increase in the concentration of flour has a positive effect on the growth of the probiotic strain, but causes a decrease in the pH of the growth medium.

The most intense growth of the strain took place when the concentration of flour in the medium was 1 and 2%. The concentration of *L. casei* was then equal to 14.39 and 15.79 log CFU/mL, respectively. Thus, it is higher than the concentration of *L. casei* cells in the control (sample without flour: 10.6 log CFU/mL) by 3.79 and 5.19 log CFU/mL, respectively. The samples mentioned above also had the lowest pH values of 4.1 (flour concentration 1%) and 3.9 (flour concentration 2%). The less intense growth rate of *L. casei* with the flour content of over 2% in an MRS medium can be associated with the increased viscosity of the medium after the addition of flour, as observed by Bustamante et al. after using the additive of flaxseed mucus.^26^

Tapioca flour is primarily composed of carbohydrates (87.5%), mainly starch, and proteins (0.1%).^27^ Thus, it can be a source of carbon during the process of growth and development of *L. casei* bacteria in addition to glucose contained in the MRS agar. The flour can also be a substrate in the biosynthesis of organic acids such as lactic, propionic, or formic acid.^26^ As the increased concentration of tapioca flour results in the presence of more sugar compounds, bacterial growth and their activity might be more affected, resulting in higher concentrations of hydrogens ion and a reduction in the pH of the MRS medium.^18^ The possibility of using enzymatically hydrolyzed tapioca flour as a source of carbon to promote growth of the *Lactococcus lactis* IO-1 (JCM7638) strain and concurrently as a substrate for lactic acid fermentation was previously suggested by Onet.^28^ Shamala and Sreekantiah proposed similar conclusions and indicated the possibility of utilizing unrefined tapioca flour in the production of lactic acid and *Lactobacillus plantarum* biomass for medical use.^29^ Based on the available data, bacteria of the *Lactobacillus* genus, especially the species *L. casei*, *L. plantarum*, and *Lactobacillus acidophilus*, have the ability to metabolize various oligosaccharides, including starch, into simple sugars and use them as a carbon source for growth and the synthesis of organic acids.^30,31^

### Results of Experimental Design for Microsphere Composition

The composition of alginate–prebiotic microspheres was optimized using mathematical methods. The viscosity, size, encapsulation efficiency, and viability after 7 days were classified as the output parameters. Table 2 shows the specific values of process composition and the results of the physicochemical analyses and viability over time of probiotic bacteria.

**Table 2:** Matrix Showing the Experimental Design for Microsphere Composition and the Experimental Data Obtained for the Dependent Variables

	independent variables				dependent variables			
sample no.	cemulsifier (%)	cprebiotic (%)	calginate (%)	mass ratio alginate to prebiotic (%)	viscosity (Pa s)	size (μm)	EE after 24 h (log CFU/g)	viability after 7 days (log CFU/g)
25	3.0	6.0	2.0	3	1.175 ± 0.12	37 ± 5	9.43 ± 0.20	9.3 ± 0.20
1	1.0	2.0	2.0	1	0.550 ± 0.04	30 ± 2	9.13 ± 0.25	8.6 ± 0.12
22	3.0	4.0	2.0	1	0.720 ± 0.04	109 ± 12	8.54 ± 0.10	2.4 ± 0.10
4	1.0	4.0	2.0	3	1.685 ± 0.10	99 ± 9	8.43 ± 0.10	0
19	3.0	2.0	2.0	2	0.910 ± 0.08	57 ± 7	7.84 ± 0.15	7.7 ± 0.12
20	3.0	2.0	3.0	1	0.580 ± 0.06	48 ± 6	8.44 ± 0.16	8.2 ± 0.15
11	2.0	2.0	3.0	2	1.155 ± 0.09	67 ± 6	8.45 ± 0.17	8.2 ± 0.15
15	2.0	4.0	4.0	3	2.823 ± 0.17	48 ± 7	9.24 ± 0.10	6.1 ± 0.10
7	1.0	6.0	2.0	2	1.123 ± 0.12	101 ± 15	9.08 ± 0.16	8.4 ± 0.12
16	2.0	6.0	2.0	1	1.304 ± 0.08	77 ± 8	8.45 ± 0.20	8.5 ± 1.12
12	2.0	2.0	4.0	1	1.661 ± 0.14	36 ± 1	9.41 ± 0.13	9.2 ± 0.10
13	2.0	4.0	2.0	2	0.783 ± 0.05	46 ± 4	9.6 ± 0.11	5.7 ± 0.15
6	1.0	4.0	4.0	1	2.751 ± 0.18	164 ± 17	9.24 ± 0.12	4.9 ± 0.13
10	2.0	2.0	2.0	3	0.929 ± 0.08	46 ± 3	8.8 ± 0.15	8.1 ± 0.18
28	2.0	4.0	3.0	2	1.322 ± 0.12	74 ± 8	9.11 ± 0.15	4.8 ± 0.12
18	2.0	6.0	4.0	2	2.617 ± 0.17	55 ± 4	8.18 ± 0.18	8.5 ± 0.10
2	1.0	2.0	3.0	3	1.480 ± 0.09	53 ± 3	8.45 ± 0.20	8.3 ± 0.15
27	3.0	6.0	4.0	1	2.890 ± 0.20	41 ± 2	8.13 ± 0.17	8.1 ± 0.11
21	3.0	2.0	4.0	3	2.617 ± 0.23	26 ± 3	7.89 ± 0.25	7.8 ± 0.25
24	3.0	4.0	4.0	2	2.760 ± 0.12	42 ± 4	8.01 ± 0.15	3.2 ± 0.10
26	3.0	6.0	3.0	2	1.580 ± 0.08	43 ± 4	8.21 ± 0.15	7.8 ± 0.12
23	3.0	4.0	3.0	3	1.720 ± 0.05	44 ± 5	8.14 ± 0.12	5.5 ± 0.15
3	1.0	2.0	4.0	2	2.513 ± 0.17	59 ± 6	9.44 ± 0.10	8.4 ± 0.11
9	1.0	6.0	4.0	3	3.290 ± 0.24	104 ± 15	7.99 ± 0.18	2.3 ± 0.10
8	1.0	6.0	3.0	1	1.155 ± 0.07	64 ± 7	9.34 ± 0.11	5.1 ± 0.14
17	2.0	6.0	3.0	3	1.627 ± 0.07	38 ± 3	8.55 ± 0.14	0
14	2.0	6.0	3.0	1	1.815 ± 0.02	56 ± 7	8.16 ± 0.15	7.2 ± 0.15
5	1.0	6.0	3.0	2	1.712 ± 0.05	42 ± 6	8.58 ± 0.20	0

**Figure 2:**
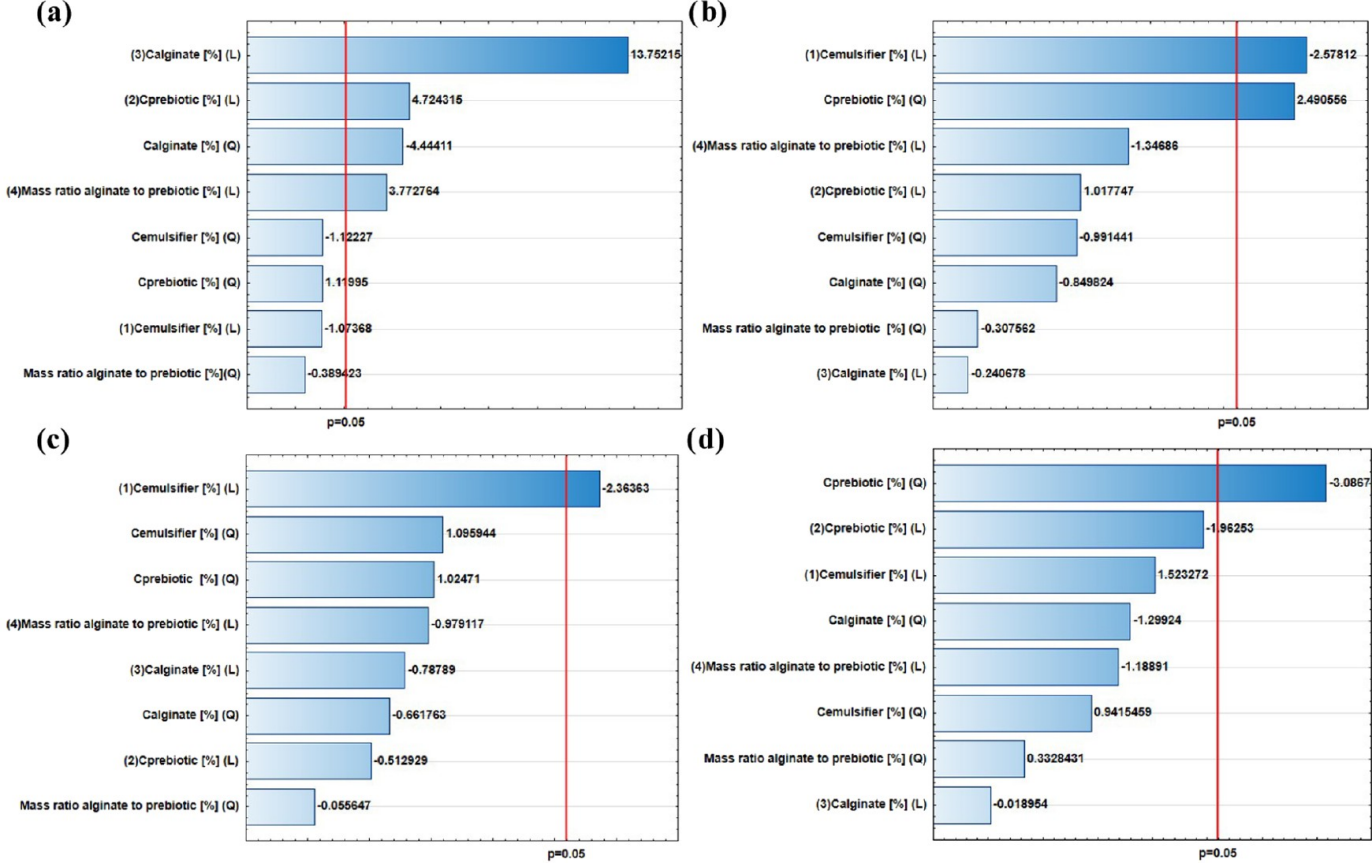
Pareto charts showing the influence of independent parameters on: (a) viscosity (Pa s), (b) size (μm), (c) encapsulation efficiency (EE) after 24 h (log CFU/g), and (d) viability after 7 days (log CFU/g).

Based on the graphs, it can be concluded that the parameters with a statistically significant influence on the viscosity of the obtained microspheres are the concentration of alginate solution as a linear and square function (indicating the strong influence of alginate concentration on viscosity), the concentration of prebiotic solution, and the mass ratio of alginate to prebiotic as a linear function (Figure 2a). Moreover, the concentration of emulsifier (linear function) and concentration of prebiotic solution (quadratic function) are parameters that have a statistically significant influence on the size of the microspheres (Figure 2b). For the input parameters of encapsulation efficiency (EE) (Figure 2c) and viability after 7 days (Figure 2d), the statistically significant parameters are the emulsifier concentration (linear function) and prebiotic solution concentration (square function), respectively.

**Figure 3:**
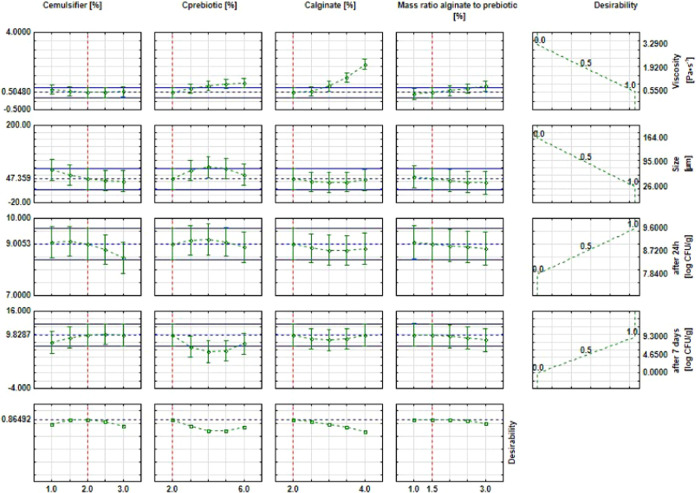
Approximation profiles for the influence of independent parameters on: viscosity [Pa•s], size [μm], encapsulation efficiency after 24 h [log CFU/g] and viability after 7 days [log CFU/g].

The next phase of the statistical analysis included the approximation profiles. The approximation profiles are presented in Figure 3 and show which values of the input parameters achieve the most desirable values of the output variables. The most desirable values are the smallest viscosity, the size of the microspheres (to ensure that bacterial encapsulation will be possible, i.e., not less than 1 μm), the highest encapsulation efficiency, and the viability of probiotic bacteria over time.

The analysis of the approximation profiles (Figure 3) shows that the microspheres with the smallest viscosity (0.550 Pa s) and size (26 μm) and the highest EE (9.6 log CFU/g) and viability over time (9.3 log CFU/g) were obtained for the lowest concentration of alginate solution (2%) and prebiotic solution (2%), intermediate emulsifier concentration (2%), and 1.5% mass ratio of alginate to prebiotic. The graphs also show that the viscosity of the microspheres increases as the concentration of alginate and prebiotic solutions and the mass ratio of alginate to prebiotic are increased. The size of the prebiotic microspheres decreases as the emulsifier concentration increases. However, the efficiency of encapsulation also decreases, which is in contrast to the expected effect. Additionally, when the mass ratio of alginate to prebiotic is increased, the EE and bacterial viability decreased over time.

**Figure 4:**
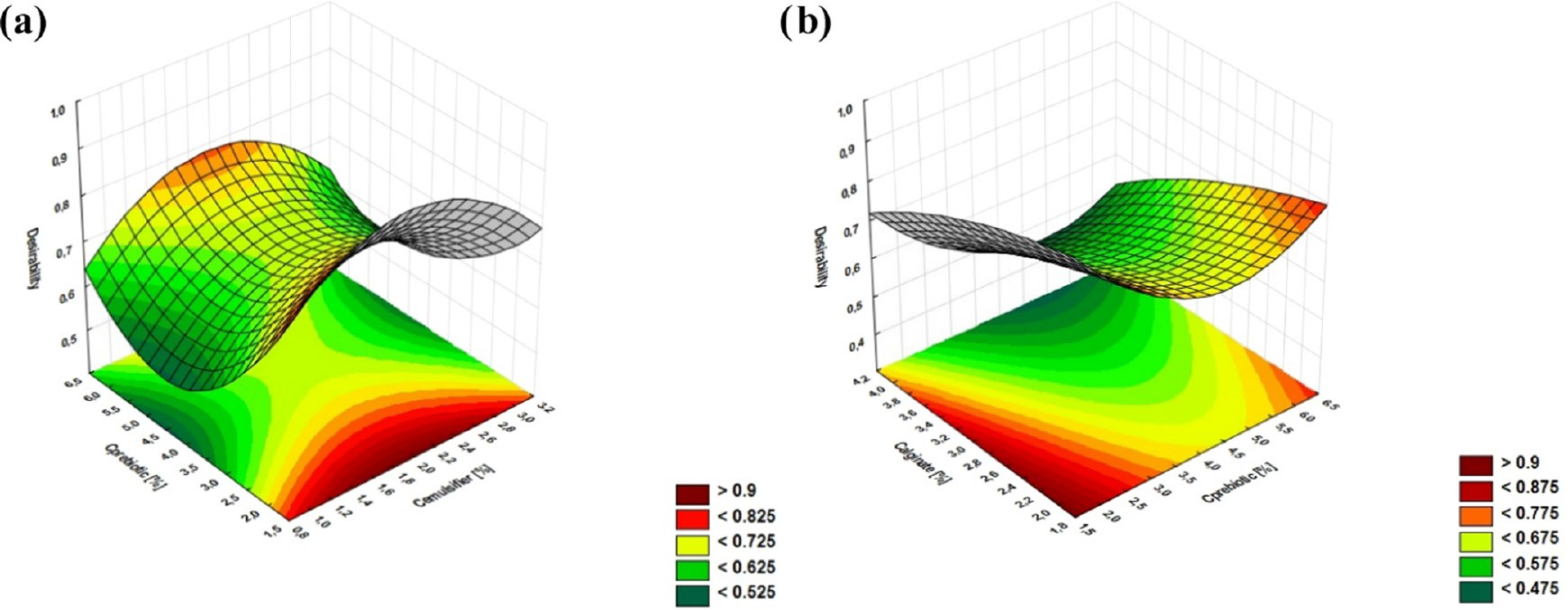
Response surface plots for the desirability with respect to (a) emulsifier and prebiotic concentration and (b) alginate and prebiotic concentration.

Figure 4 shows the surface response plots for the most desirable microsphere composition, i.e., emulsifier and prebiotic concentrations, and alginate and prebiotic concentrations; the range was extended by model calculations.

The data presented in Figure 4 show that to obtain the microspheres with the desired parameters, such as the smallest droplet size and the lowest viscosity, and the highest EE and viability over time of probiotic bacteria, the concentrations of the emulsifier and prebiotic solution should be in the range of 1.0 to 2.5% and below 1.5%, respectively. However, in the case of the influence of the concentrations of prebiotic and alginate solutions on desirability, the most optimal values were in the ranges of 1.8–2.2 and 1.5–1.6%, respectively.

**Figure 5:**
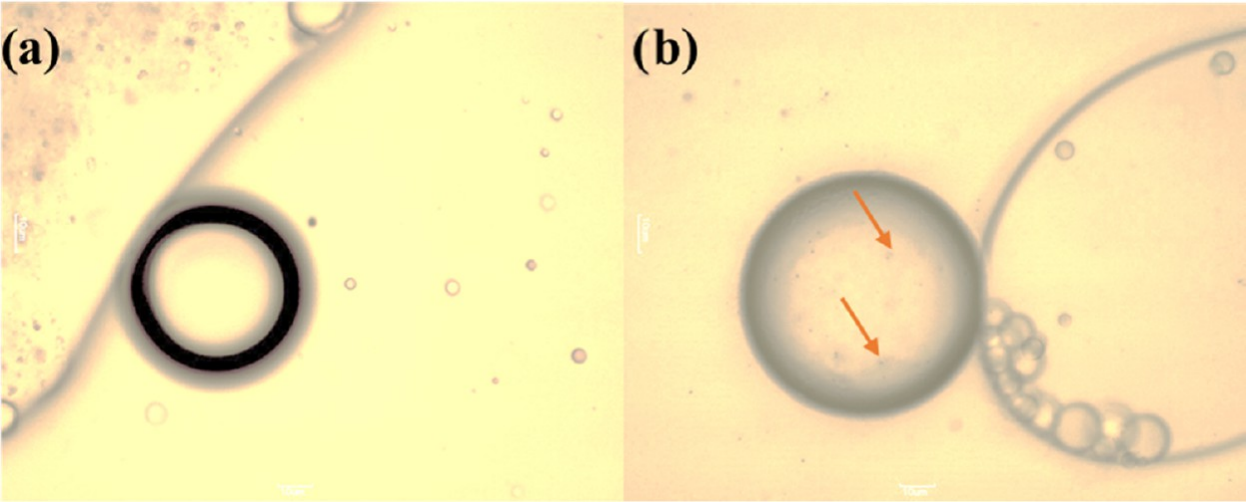
Alginate–prebiotic microspheres containing *L. casei* probiotic before freeze-drying, (a) structure of microsphere, (b) microsphere with encapsulated *L. casei*.

Based on the design of experiments (DOE) results, the alginate–prebiotic microspheres (Table 3) were prepared as well as other coatings. Additionally, optical micrographs of alginate–prebiotic microspheres containing probiotic *L. casei* are shown in Figure 5.

**Table 3:** Input and Output Parameters for the Optimal Composition of Alginate–Prebiotic Microspheres

cemulsifier (%)	cprebiotic (%)	calginate (%)	mass ratio alginate to prebiotic (%)	viscosity (Pa s)	size (μm)	EE after 24 h (log CFU/g)	viability after 7 days (log CFU/g)
1.0	2.0	2.0	1.5	0.530 ± 0.1	30.00 ± 4.25	9.31 ± 0.2	9.06 ± 0.4

### SEM Analysis

**Figure 6:**
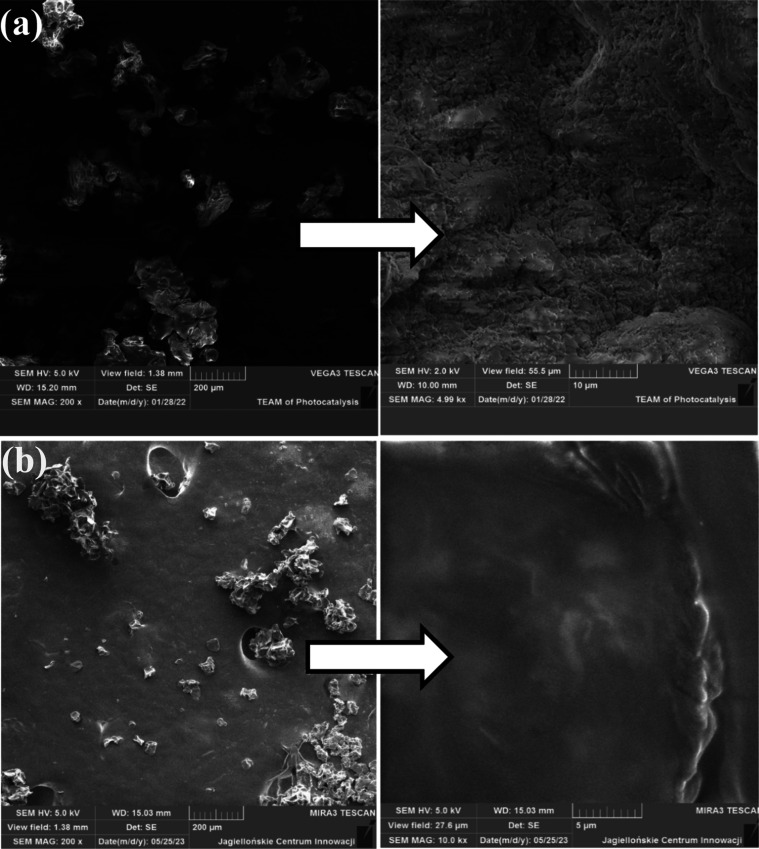
SEM micrographs of cross sections of freeze-dried (a) alginate microspheres (reference sample) and (b) alginate–tapioca flour microspheres.

The surface morphology and cross-section of alginate microspheres reference sample^13^ and alginate–prebiotic microspheres were analyzed using scanning electron microscopy (SEM) (Figure 6).

The alginate microspheres (Figure 6a) were generally spherical with a wrinkled surface.^11,32^ The wrinkled surface was probably a result of the water content lost during the freeze-drying process.^33−35^ Dolly et al.^36^ reported that during the lyophilization of polysaccharide hydrogel spheres, ice crystals were formed at the low temperatures to which the spheres were exposed to during preparation for freeze-drying. After sublimation of the ice crystals under reduced pressure, a porous, dry, and spongy matrix is formed. In turn, Fareez et al.^37^ linked surface irregularities of the particles to higher concentrations of polymer in specific regions of the closed spheres. The addition of tapioca flour did not change the shape or size of the microparticles (Figure 6b). However, the addition of a prebiotic source had a positive effect on the modification of the surface of the microspheres; consequently, the porous structure was smoothed and the alginate microparticles became more sealed.

**Figure 7:**
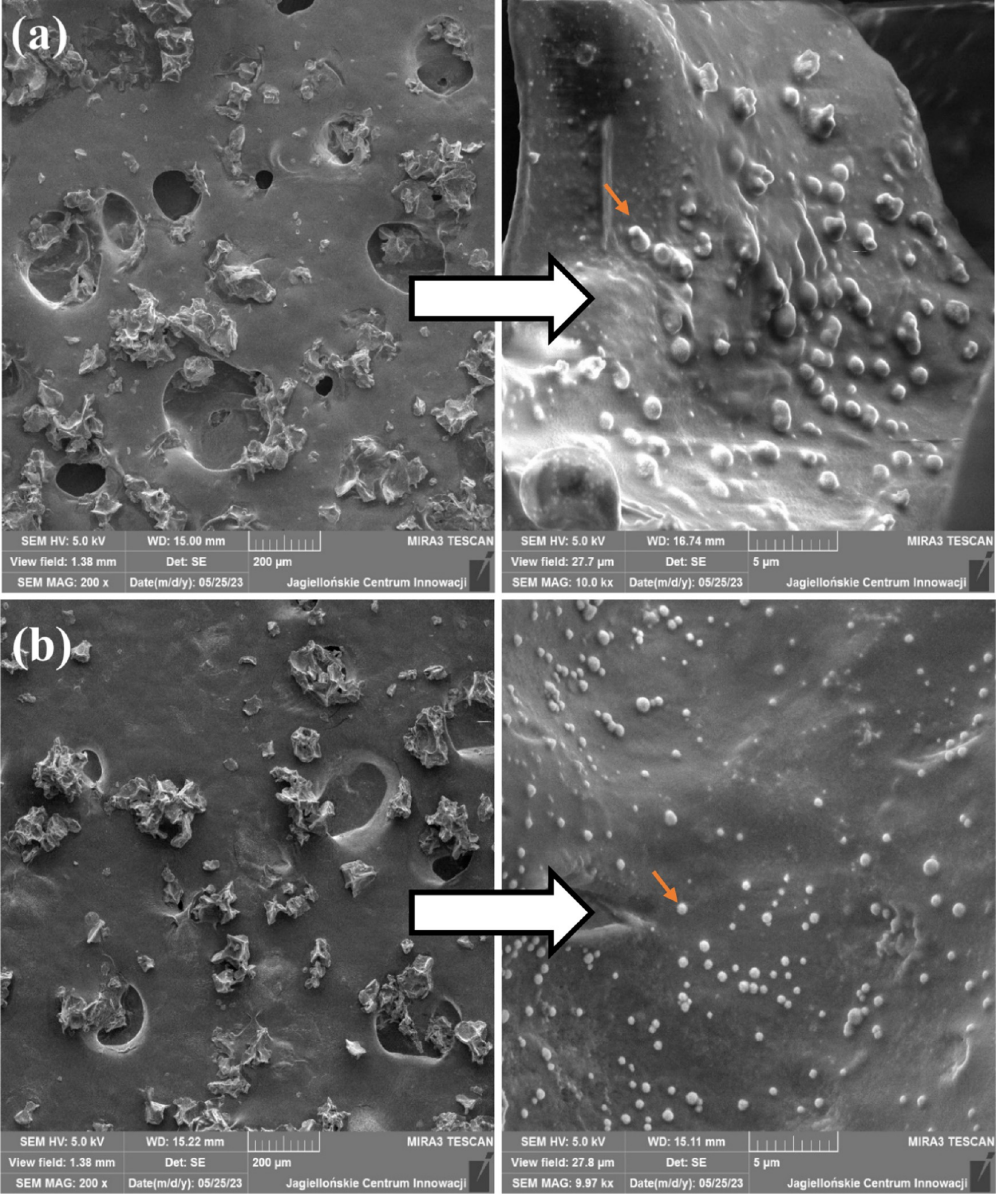
SEM micrographs of the structure of freeze-dried (a) alginate microspheres with encapsulated *L. casei* and (b) alginate–tapioca flour microspheres with encapsulated *L. casei*.

The structure of alginate and alginate–prebiotic microspheres with encapsulated *L. casei* is presented in Figure 7. As can be seen, in both cases (Figure 7a,b), microorganisms were present in the sphere (marked with an arrow).

### FTIR Analysis

**Figure 8:**
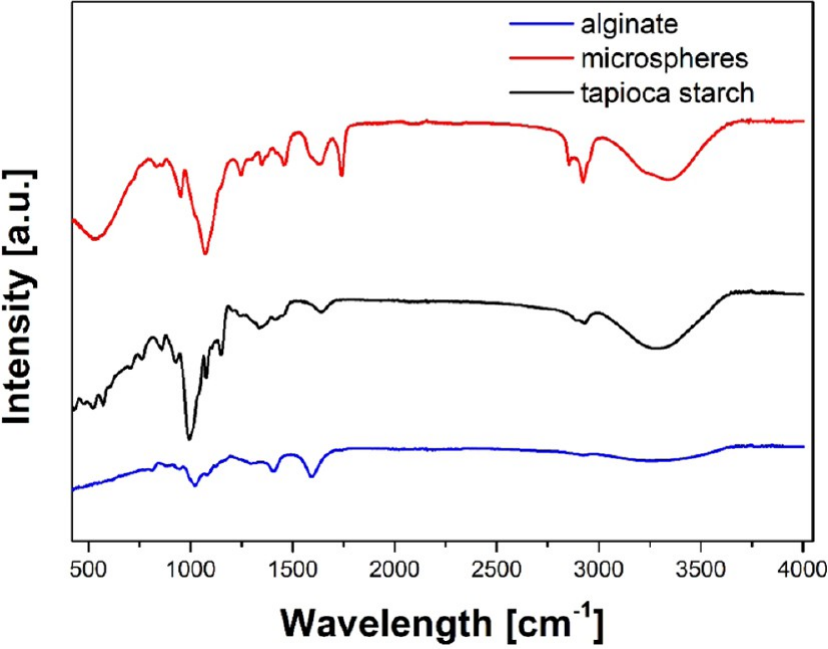
FTIR of plain sodium alginate, plain tapioca flour (prebiotic source), and alginate–prebiotic microspheres with *L. casei*.

The FTIR spectra of alginate, tapioca flour, and *L. casei*-containing microsphere were used to detect functional groups that may illustrate various interactions between molecules (Figure 8).

A broad peak at approximately 3252 cm^–1^ appears in the plane of the sodium alginate powder, which relates to the O–H stretching vibration. The peak near 1592 cm^–1^ is related to the asymmetric stretching of the −C=O of the carboxyl group, and the peak near 1405 cm^–1^ is related to the symmetrical stretching of the COO group. The peaks at approximately 1020 cm^–1^ correspond to the hydrogen and C–H bonds, respectively. The vibration absorption bands at 1000–850 cm^–1^ arise from the C–H bonds of the monosaccharides.^38,39^

In the spectrum of tapioca flour, characteristic FTIR absorption peaks were observed, e.g., at 927, 993, 1149, 1335, and 3287 cm^–1^. The infrared absorption at approximately 925 cm^–1^ was attributed to glycosidic bonds in starches and asymmetric C–O–C stretching.^40^ The peak at 993 cm^–1^ was associated with the C–OH bending vibration. The absorption peak at 1149 cm^–1^ was probably a result of the C–O and C–C stretching coupling, whereas the peak at 1076 cm^–1^ could be attributed to the C–O–H bending mode. Moreover, the slight peaks between 1149 and 1076 cm^–1^ may be due to the amylopectin content of the starch granules, which depends on the type of starch. In addition, the infrared band at 1335 cm^–1^ may have originated from CH_2_ bending. The FTIR spectra of starch show the C–H stretching mode in the range of 2800–3000 cm^–1^ and the O–H stretching mode in the range of 3000–3600 cm^–1.^^41,42^ The O–H stretching mode of starch was observed at 3287 cm^–1^ whereas the C–H stretching mode was approximately 2929 cm^–1^.

FTIR spectra of alginate–tapioca flour microspheres with entrapped probiotic cells contained slightly shifted, typical polysaccharide vibrational bands with characteristic alginate bands in the regions of 3224, 1456, and 1072 cm^–1^. The spectrum also includes bands typical of tapioca flour: 2934, 1632, and 1072 cm^–1^. A broad and strong absorption band appears at approximately 3600–3200 cm^–1^ in both composite materials and is slightly shifted in the microspheres containing probiotic bacteria, suggesting that hydrogen bonds may play an important role in the formation of the biocomposite. The new sharp peak at 1740 cm^–1^ may be related to the amide bonds found in probiotic cellular proteins.^17^ The intense bands at 1740 and 1632 cm^–1^ are also associated with amide band I (C=O stretching vibrations) of functional groups from endogenous proteins and amide band II (angle deformation C–N–H in the plane and C–N segment of probiotic cellular proteins).^17,32^ The ATR-FTIR spectrum of probiotic-loaded microspheres contained a peak at ∼1072 cm^–1^, which was identified in the literature as stretching vibrations from the phosphoric acid groups in the nucleic acids. Absorption in the range from 1200 to 900 cm^–1^ can be attributed to symmetrical stretching vibrations of the phospho-oxygen (P–O) phosphodioxy group (PO_2_−), as well as deformation vibrations of C–O–C of polysaccharides belonging to the glycoproteins of the cell membrane and lipopolysaccharides of the membrane of probiotic cells.^32,43^ The band at 831 cm^–1^ is in the region of 900–700 cm^–1^. This region is considered the true fingerprint region because it contains very specific and weak spectral patterns showing the vibrations of the aromatic ring of aromatic amino acids (tryptophan, tyrosine, phenylalanine) and nucleotides.^44^ Absorption in this region is related to the presence of cellular material in the encapsulated probiotic cells.

### Coating of Alginate–Prebiotic Microspheres

To encapsulate living cells, a neutral pH is most appropriate, as it does not damage the cellular structures. However, acid-tolerant cultures, such as lactic acid bacteria, can be immobilized in the lower pH range, down to pH 5.^11,45^ Moreover it should be noticed that despite the addition of a prebiotic, the porous structure of microspheres was smoothed and the alginate microparticles were sealed, showing that tapioca flour is the most useful food ingredient for encapsulating prebiotics and stimulating their growth. Therefore, solutions of biopolymers with slightly acidic pH, such as gelatin, chitosan, and hyaluronic acid, were prepared to coat the microspheres and further seal their structure. Table 4 shows the pH of the solutions used to coat the microspheres containing *L. casei* bacteria and the particle size distribution of the resulting microspheres.

**Table 4:** pH of Coating Solutions and the Particle Size Distribution of Microspheres

coating material	pH	particle size [μm]
uncoated		30.1 ± 2.2
chitosan	5.73	37.5 ± 3.6
gelatin	5.36	32.6 ± 2.1
hyaluronic acid	5.51	34.6 ± 1.6

The mean diameter of the microspheres without the additional coating layer was 30.1 ± 2.2 μm. The additional coating agents slightly increased the size of the microspheres. This is consistent with other literature reports.^46^ In our case, a slight increase in the size of the spheres resulted from the coating. Koo et al.^47^ reported that the shape and size of the beads did not change significantly when chitosan was added as a coating agent to alginate beads. Micron-sized spheres have been prepared in several reports to provide a soft texture when added to other products such as food.^48,49^ In other reports of spheres obtained by the emulsification technique, the sizes are much larger (>100 μm). The mean diameter of microspheres without additional chitosan coating was 92 ± 1.709 μm in the study of Zanjani et al.,^21^ whereas that of chitosan-coated microspheres was much larger, at 124 ± 1.96 μm. In our study, the obtained microparticles had a size of less than 40 μm, despite the biopolymer coating.

### Raman Spectroscopy Analysis

Raman images (usually 50 × 50 μm^2^ in size) were collected with a sampling density of 1 μm in the *x*, *y* plane and a spectral accumulation time of 0.5 s. Within each sample, Raman images were recorded, which were then subjected to routine processing consisting of removing cosmic rays and correcting the baseline of the spectra. In the next step, a chemometric analysis (*k*-means cluster analysis, KMC) was performed to group similar spectra into classes. In the KMC analysis, pixels within the same class were coded with the same color, and the spectra of a given class were averaged. The background area (outside the microcapsule) is coded in black.

**Figure 9:**
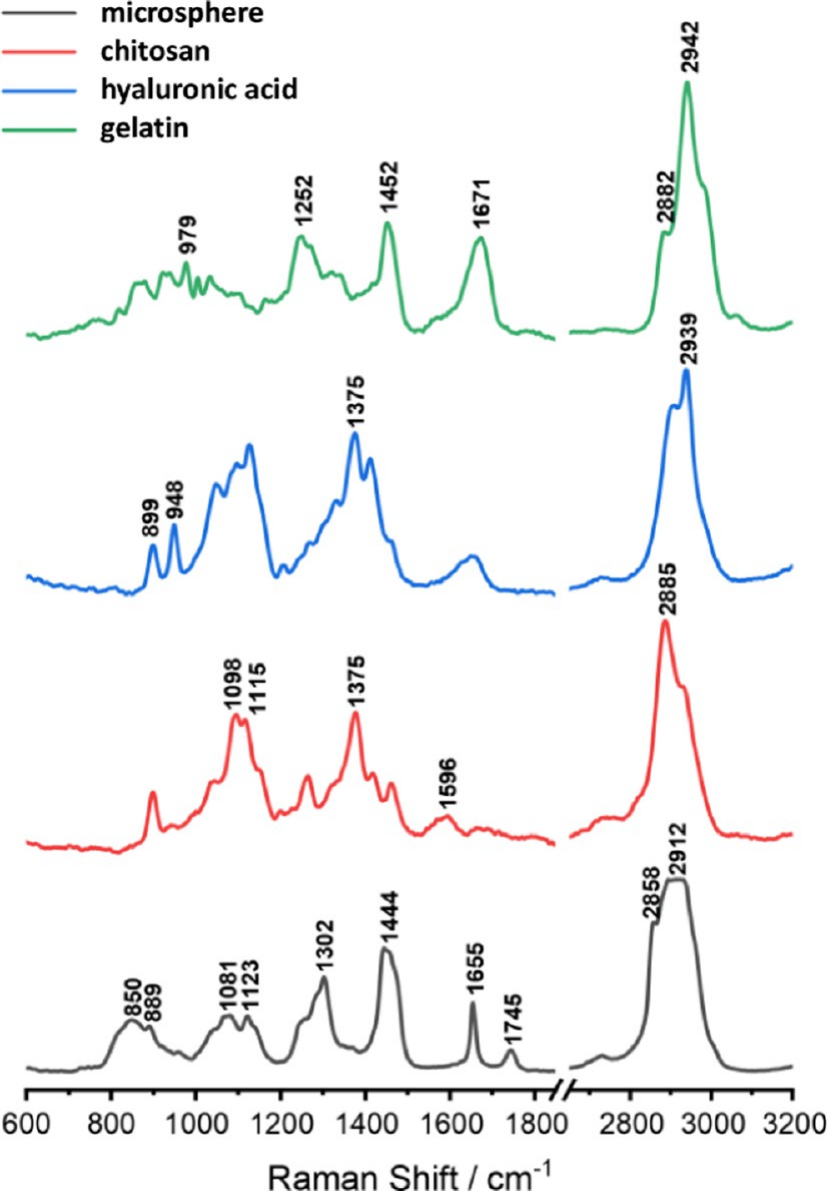
Comparison of the average spectra of standards of hyaluronic acid, chitosan, and gelatin with the average spectrum of uncoated microspheres. The characteristic bands are marked on the spectra of the standards.

Figure 9 presents the averaged spectra of the standards along with the average spectrum recorded for the sample containing uncoated microspheres. In the average spectrum of uncoated microspheres, there are intense and well-separated bands at positions 1302, 1444, and 1655, and a wide band in the range from 2800 to 3050 cm^–1^. In the case of standard spectra, marker bands are indicated in Figure 9, which could be used to identify them in the analysis of coated microspheres (as these bands are not present in the spectrum of uncoated microspheres). Hyaluronic acid can be identified based on the bands found at positions 899, 948, and 1375, whereas chitosan is identified mainly on the band at 1375 cm^–1^. Gelatin is characterized by a set of bands at 1252, 1452, 1671, and 2942 cm^–1^. Owing to the overlapping of the bands in the so-called “high range” (2800–3050 cm^–1^) for the spectra of standards and uncoated microspheres, this region was not taken into account during the analysis.

**Figure 10:**
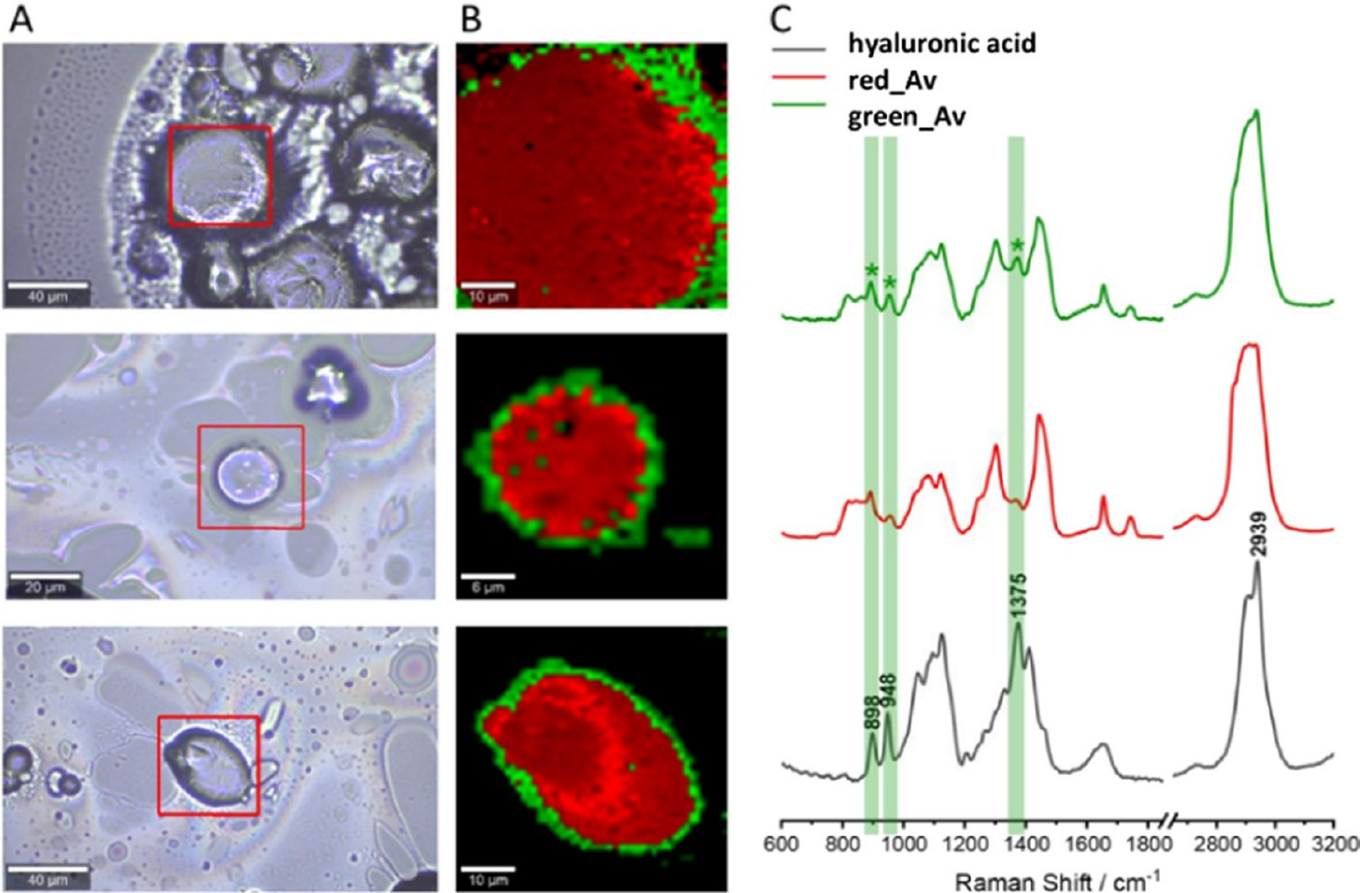
Raman mapping results for selected microspheres coated with hyaluronic acid: images with measurement areas marked (A) with KMC analysis (B) and average spectra for classes (spectral colors correspond to class colors in KMC images) compared with the standard spectrum (C). Bands indicating the presence of hyaluronic acid on the surface of the microspheres are marked as a green area.

Raman images for coated microspheres were collected for well-isolated objects that were approximately spherical and without visible damage. In the case of all tested microspheres coated with hyaluronic acid (Figure 10), the KMC analysis enabled the separation of two classes within the studied objects, one covering the greater part of the mapped surface containing the central part of the microsphere (colored red) and the other located on the edges (coded in green). The spectrum of the class separated at the edges of the microspheres contains distinct bands at approximately 890, 950, and 1375 cm^–1^, which may be attributable to hyaluronic acid (they are not present in the spectrum recorded for the sample containing uncoated microspheres). The red class spectrum also contains these bands, but they are less intense, which is related to the focal plane and the volume from which the spectrum is collected in each pixel.

**Figure 11:**
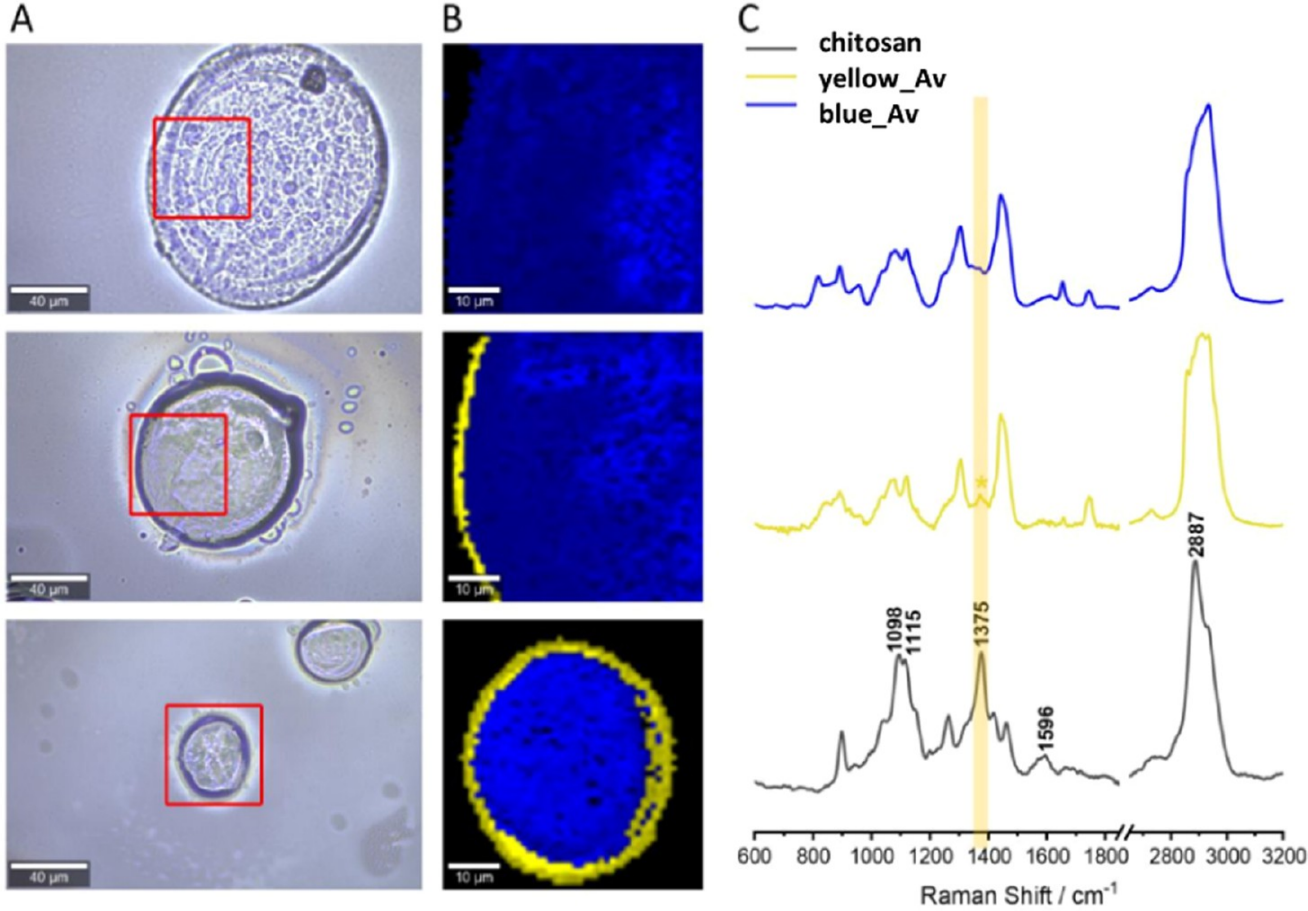
Raman mapping results for selected chitosan-coated microspheres: images with measurement areas marked (A) with KMC analysis (B) and average spectra for the classes (the colors of the spectra correspond to the colors of the classes in the KMC images) compared with the standard spectrum (C). A band indicating the presence of chitosan on the surface of the microspheres is marked in yellow.

Similar results were obtained for chitosan-coated microspheres (Figure 11). In the case of two out of three objects, the chemometric analysis revealed two classes with different spectral profiles: the boundary layer (colored yellow) and the larger-area middle layer (coded in blue). The spectrum of the blue class is similar to the spectrum from the sample containing uncoated microspheres; it is difficult to find bands indicating the presence of chitosan. However, the spectrum of the yellow class (the edge of the microspheres) has a low intensity band located at 1375 cm^–1^. This band is also present in the chitosan standard spectrum, which may indicate the presence of this compound on the surface of two of three imaged microspheres.

**Figure 12:**
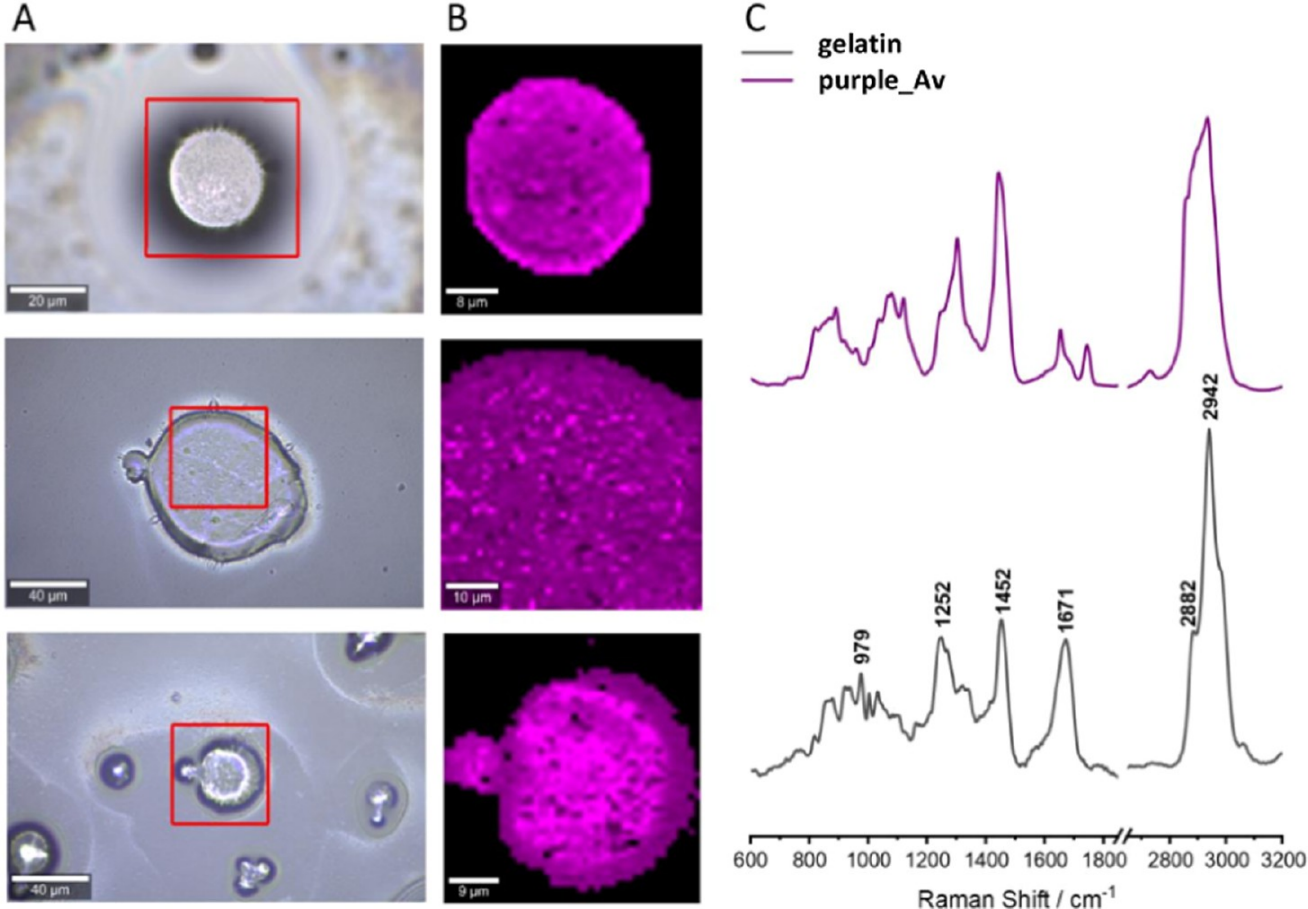
Raman mapping results for selected gelatin-coated microspheres: images with measurement areas marked (A) along with KMC analysis (B) and the average spectrum of the microsphere compared to the gelatin standard (C). In the microspheres spectra, there are no visible bands indicating the presence of gelatin on their surface.

The third sample tested contained gelatin-coated microspheres (Figure 12). In the case of this sample, owing to the high similarity of the spectra recorded within the tested objects, only one class was distinguished based on the KMC analysis. The average spectrum from this class is similar to the spectrum recorded for the sample of uncoated microspheres; there are no visible bands characteristic of the gelatin standard spectrum.

The bacteria (size 1–3 μm) are retained quite well in the alginate gel matrix. However, these microspheres have a porous structure (Figure 6a). The addition of tapioca flour reduces the pores and smooths the surface of the microspheres (Figure 6b). Conversely, alginate microparticles are susceptible to disintegration in the presence of excess monovalent ions, Ca^2+^ chelating agents, and harsh chemical environments.^50^ It is reported that the cross-linked alginate matrix system at low pH reduces the molecular weight of alginate, causing faster degradation and release of active ingredients.^20^ Therefore, the use of another anionic agent coating the surface of the microspheres (in our case, hyaluronic acid) can prevent the degradation of alginate structures, thereby contributing to the increased stability of the microspheres under the influence of unfavorable conditions. Additionally, the use of tapioca flour may reduce the repulsion between polymers and thus improve the cross-linking of the coated structures.

Coating alginate microspheres with chitosan is well established in the scientific literature. Dropping alginate solution into a solution containing a mixture of calcium chloride and chitosan^51^ or soaking alginate beads in this solution^52^ creates chitosan-coated alginate beads, resulting in a polyelectrolyte complex. In the research of Zhou et al.,^53^ the use of chitosan coating reduced cell release by 40%.

The advantages of gelatin include its ability to form membranes, probiotic biocompatibility, and nontoxicity. Additionally, gelatin and sodium alginate can form a strong complex arising from the electrostatic interactions between the amide group of gelatin and the carboxyl groups of alginate.^54^ However, in the case of gelatin, the effectiveness of the coating cannot be clearly determined. The peaks are at similar lengths with respect to uncoated microspheres, but a second peak is visible at a length of approximately 1671 cm^–1^. In some reports, gelatin is described with a characteristic peak at 1639 cm^–1^,^55^ so the spheres may also be coated with gelatin, but this cannot be confirmed. Additionally, the pH of the coating solution could have influenced the quality of the coating. The isoelectric point of gelatin is approximately 7–9; thus, under neutral pH conditions, it has a positive charge,^56^ and the coating solution used has a slightly acidic pH, which may also affect the quality of the coating.

### Survival of the Encapsulated *L. casei* Strain During Storage

Literature studies indicate that encapsulation of probiotic bacteria leads to significantly higher viability compared with free cells. In the study conducted by Dimitrellou et al.,^57^ the strain *L. casei* ATCC 393 was encapsulated in alginate capsules for the production of probiotic fermented milk. As a result, high bacterial survival (7.13 log CFU g^–1^) was obtained as a result of storing the product at 4 °C for 4 weeks. The results are in agreement with those of other research groups. Karkar and co-workers^58^ encapsulated the probiotic bacteria *L. casei* and *L. acidophilus* in oleaster flour, which is rich in phenolic compounds and has potential prebiotic properties, and then stored at −24 °C for 28 days. As a result of storage, the viability for *L. casei* bacteria was less than 20%. Similar results were also obtained by Hadzieva et al.^59^*L. casei* bacteria were encapsulated in a soy protein isolate and sodium alginate. As a result of microencapsulation, the survival rate of *L. casei* was 82% during storage for 4 months at 4 °C. However, the survival studies over time were conducted at low temperatures (4 and −24 °C).

Therefore, to increase the survival of probiotic strains at room temperature, in addition to the prebiotic source, additional coating of the spheres can be used.^60^ Such biopolymers include chitosan,^52,61^ poly-l-lysine,^20,62^ gelatin,^46,55^ Eudragit L100-55,^61^ Eudragit S100,^63^ maltodextrin,^64^ and collagen.^65^ Therefore, we aimed to determine whether the addition of tapioca flour at a concentration of 2% as a source of prebiotic and the use of coating with chitosan, gelatin, or hyaluronic acid significantly affected the survival of the *L. casei* strain and its viability in spheres during storage at room temperature.

**Figure 13:**
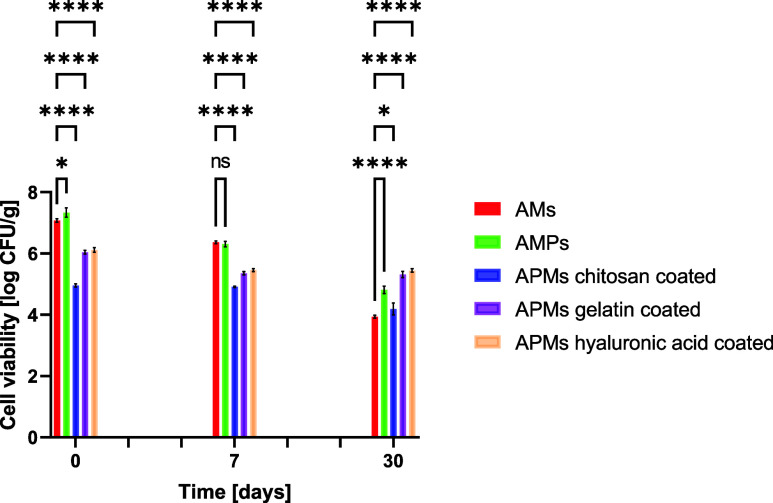
Comparison of the viability of encapsulated *L. casei* in alginate microspheres (reference sample), alginate–prebiotic microspheres (AMPs), and AMPs coated with chitosan, gelatin, and hyaluronic acid during 30 days of storage at room temperature. *p = 0.05–0.011; **p ≤ 0.01; ***p ≤ 0.001; ****p ≤ 0.0001.

In the first stage of the study, the survival of the *L. casei* strain encapsulated in alginate microspheres (AMs), alginate microspheres enriched with prebiotics (AMPs), and alginate microspheres with prebiotics additionally covered with various substances (APMs chitosan, APMs gelatin, APMs hyaluronic acid) was assessed. Survival was assessed immediately after capsule production and after 7 and 30 days of storage, and the results obtained were expressed as log CFU g^–1^ (Figure 13).

The initial density of the *L. casei* strain suspension used to obtain spheres was 8.1 log CFU g^–1^. Immediately after receiving the spheres, the highest survival of the *L. casei* strain was observed for APMs and AMs spheres, of 7.31 and 7.01 log CFU g^–1^, respectively. Slightly lower survival values were observed for APMs spheres coated with other substances: 4.97 log CFU g^–1^ (APMs chitosan coated), 6.11 log CFU g^–1^ (APMs gelatin coated), and 6.05 log CFU g^–1^ (APMs hyaluronic acid coated).

After 7 days of storage at room temperature, the survival rate of the probiotic strain decreased to 6.32 log CFU g^–1^ in APMs spheres and 6.22 log CFU g^–1^ in AMs spheres. For the remaining spheres, the survival rates of *L. casei* were 4.41 log CFU g^–1^ (APMs chitosan coated), 5.37 log CFU g^–1^ (APMs gelatin coated), and 5.27 log CFU g^–1^ (APMs hyaluronic acid coated).

After 1 month of storage, a significant change in the survival of the *L. casei* strain was compared with the results of fresh spheres and after 7 days of storage. The highest survival rate of the probiotic strain was recorded for APMs hyaluronic acid-coated spheres, of 5.48 log CFU g^–1^. Slightly lower strain survival was found in APMs gelatin-coated spheres of 5.34 log CFU g^–1^. In the case of the remaining spheres, the obtained survival values were as follows: 3.99 log CFU g^–1^ (AMs), 4.84 log CFU g^–1^, and 4.94 log CFU g^–1^ (APMs chitosan coated).

**Figure 14:**
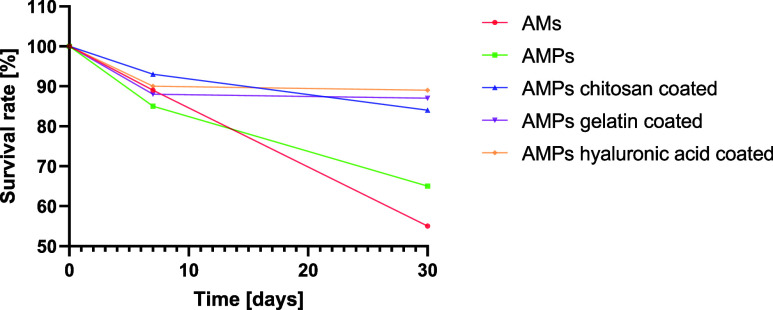
Survival rates of *L. casei* in AMs, AMPs, AMPs chitosan-, gelatin-, and hyaluronic acid-coated microspheres over 30 days of storage at room temperature.

We also assessed how the percentage viability of the strain encapsulated changed after 7 and 30 days of storage compared to the viability immediately after sphere production (100% viability) (Figure 14).

After 7 days of storage, the highest viability of the *L. casei* strain was found in the case of APMs chitosan coated, APMs hyaluronic acid coated, and AMs spheres, with values of 93.8, 90.4, and 89.9%, respectively. The viability of the strain in other spheres was slightly lower (88.6% for APMs gelatin-coated spheres and 86% for AMPs spheres).

Extending the storage period to 30 days resulted in a 10.96% reduction in the viability of the *L. casei* strain in the case of APMs hyaluronic acid-coated spheres and a 12.1% reduction in the case of APMs gelatin-coated spheres compared with the initial value. In the remaining spheres, a much higher reduction in survival values was found: 16.81% (APMs chitosan coated), 34.4% (AMPs), and 44.4% (AMs).

The obtained results therefore indicate that the addition of a prebiotic and the coating of microspheres with gelatin and hyaluronic acid significantly affected the survival rate (survival rate of 5.48–5.34 log CFU g^–1^) as well as maintaining the viability of the probiotic strain (viability rate of 89–87.9%) during long-term storage. The effect of chitosan was slightly less effective than hyaluronic acid or gelatin. The obtained strain survival results for chitosan-coated spheres after 1 month of storage were similar to those for prebiotic spheres (survival of 4.84–4.94 log CFU g^–1^). The use of chitosan coating allowed the viability of *L. casei* to be maintained at 83.2%; for probiotic spheres, this value was 65.6%.

The effectiveness of gelatin coating was already reported by da Conceição et al.^54^ They encapsulated two probiotic strains of *Lactobacillus paracasei* (LBC 81 and ELBAL) with fructooligosaccharides (FOS) as a prebiotic in a calcium alginate matrix using extrusion technology with gelatin as a coating material. Both strains were characterized by high viability under the tested stress conditions, such as a simulated gastrointestinal environment and low-temperature storage. Previous research has also shown that an alginate matrix coated with gelatin can improve the survival of probiotic bacteria in unfavorable conditions.^66,67^

Several studies have shown that low-molecular-weight chitosan is effective as a microcapsule coating agent despite its antimicrobial properties.^68,69^

The research of Erdélyi et al.^61^ into the development of a probiotic preparation for animals in the form of chitosan-coated microspheres containing strains of the genus *Bifidobacterium* and *Lactobacillus* showed lower resistance to stress conditions (heat) and lower viability of the strains compared with uncoated microspheres. Our research has shown that coating microspheres with chitosan can increase the viability of the *L. casei* strain without significantly affecting its survival over 30 days of storage. It should therefore be noted that encapsulating different strains of bacteria can present different behaviors.^70^ Coating alginate beads with chitosan has been found to create a complexation between the two materials, resulting in important properties such as reduced porosity, decreased encapsulated bacteria leakage, and high stability across varying pH ranges. This is because the negatively charged alginate interacts with the positively charged chitosan, forming a semipermeable membrane.^71^

According to available literature data, no work has examined the use of hyaluronic acid to coat AMs containing probiotic bacteria. However, hyaluronic acid has been used as a substance for coencapsulation of AMs for potential use as vehicles for drug delivery to the lungs.^39,72^ In the study by Ratanavaraporn et al.,^56^ the use of hybrid alginate/hyaluronic acid spheres as a carrier for gentamicin was also described, which resulted in sustained release of the antibiotic. Moreover, in the work of Cañibano-Hernández et al.,^73^ hybrid alginate–hyaluronic acid microspheres were used to encapsulate insulin-producing cells. According to their study, the inclusion of hyaluronic acid in AMs resulted in an increase in the viability of insulin-producing pancreatic islet cells, reducing the percentage of cells displaying early apoptosis and membrane damage. The studies presented in this article showed that hyaluronic acid has the greatest impact (89%) on improving the viability of *L. casei* during storage.

The differences in the survival and viability of probiotic bacteria in microspheres coated with hyaluronic acid, gelatin, and chitosan may result from the antibacterial properties of chitosan. Chitosan and chitosan derivatives have a killing effect on various species of microorganisms by neutralizing the negative charges on their surface.^74,75^ According to No,^75^ MIC values of chitosan with molecular weights in the range from 224 to 28 kDa for three strains of probiotic bacteria of the *Lactobacillus* genus were as follows: *L. plantarum* (0.05–0.05%), *Lactobacillus brevis* (>0.1–0.08%), and *Lactobacillus bulgaricus* (0.1–0.1%).

## Conclusions

In this study, alginate–tapioca flour microspheres coated with different biopolymers, such as hyaluronic acid, chitosan, and gelatin, were obtained. The use of microencapsulation techniques, such as emulsification, obtained microcarriers that were smaller than 40 μm in size. The addition of prebiotics and biopolymer coating of the microspheres, especially with hyaluronic acid and chitosan, made their structure more smooth and sealed, which strongly affected the survival and viability of the encapsulated probiotic strain (*L. casei* bacteria) during long-term storage. The highest survival rate of the probiotic strain was recorded for alginate–tapioca flour microspheres covered with hyaluronic acid and maintained the viability of *L. casei* at 89% during storage for 30 days compared with a value of 65.6% for uncoated probiotic spheres. Considering these promising results, microcarriers of live bacteria are significant for use in cosmetic products. The use of probiotics in topical preparations can be an alternative or supplement for skin affected by inflammation, including atopic dermatitis or dry or sensitive skin. Restoring the balance of the bacterial microflora of the skin will translate into the proper functioning of the skin barrier and the reduction of inflammation related to the disruption of skin barrier integrity and the multiplication of pathogenic bacteria. Therefore, further *in vitro* and *in vivo* studies are necessary to confirm the potential benefits for the skin.
